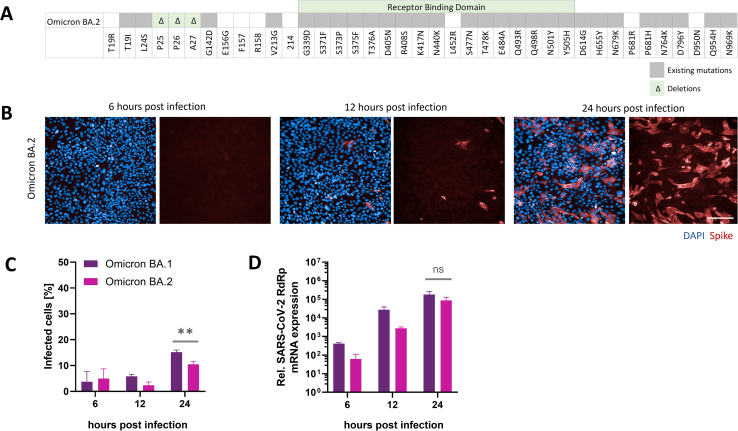# SARS-CoV-2 Variants Show Different Host Cell Proteome Profiles With Delayed Immune Response Activation in Omicron-Infected Cells

**DOI:** 10.1016/j.mcpro.2023.100537

**Published:** 2023-03-30

**Authors:** Melinda Metzler, Rebecca George Tharyan, Kevin Klann, Katharina Grikscheit, Denisa Bojkova, Jindrich Cinatl, Georg Tascher, Sandra Ciesek, Christian Münch

**Affiliations:** 1 Institute of Medical Virology, University Hospital Frankfurt, Goethe University, Frankfurt, Germany; 2 Institute of Biochemistry II, Faculty of Medicine, Goethe University, Frankfurt, Germany; 3 Fraunhofer Institute for Translational Medicine and Pharmacology (ITMP), Frankfurt, Germany; 4 German Center for Infection Research, DZIF, External Partner Site, Frankfurt, Germany; 5 Frankfurt Cancer Institute, Goethe University, Frankfurt, Germany; 6 Cardio-Pulmonary Institute, Goethe University, Frankfurt, Germany

**Keywords:** Proteomics, Covid-19, SARS-CoV-2, Omicron, Interferon, NF-kB, IRF-3, Protein-translocation

## Abstract

The ancestral SARS-CoV-2 strain that initiated the Covid-19 pandemic at the end of 2019 has rapidly mutated into multiple variants of concern with variable pathogenicity and increasing immune escape strategies. However, differences in host cellular antiviral responses upon infection with SARS-CoV-2 variants remain elusive. Leveraging whole-cell proteomics, we determined host signaling pathways that are differentially modulated upon infection with the clinical isolates of the ancestral SARS-CoV-2 B.1 and the variants of concern Delta and Omicron BA.1. Our findings illustrate alterations in the global host proteome landscape upon infection with SARS-CoV-2 variants and the resulting host immune responses. Additionally, viral proteome kinetics reveal declining levels of viral protein expression during Omicron BA.1 infection when compared to ancestral B.1 and Delta variants, consistent with its reduced replication rates. Moreover, molecular assays reveal deferral activation of specific host antiviral signaling upon Omicron BA.1 and BA.2 infections. Our study provides an overview of host proteome profile of multiple SARS-CoV-2 variants and brings forth a better understanding of the instigation of key immune signaling pathways causative for the differential pathogenicity of SARS-CoV-2 variants.

## Highlights

• Omicron variants display attenuated replication kinetics in Calu-3 cells.
• SARS-CoV-2 variants differently alter global host proteome landscapes.
• Infection with SARS-CoV-2 variants elicits shared enrichment in host immune responses.
• SARS-CoV-2 variants examined in this study induce IFN and NF-kB signaling.
• Delayed host immune response activation upon infection with Omicron variants.

## Graphical Abstract

**Figure:**
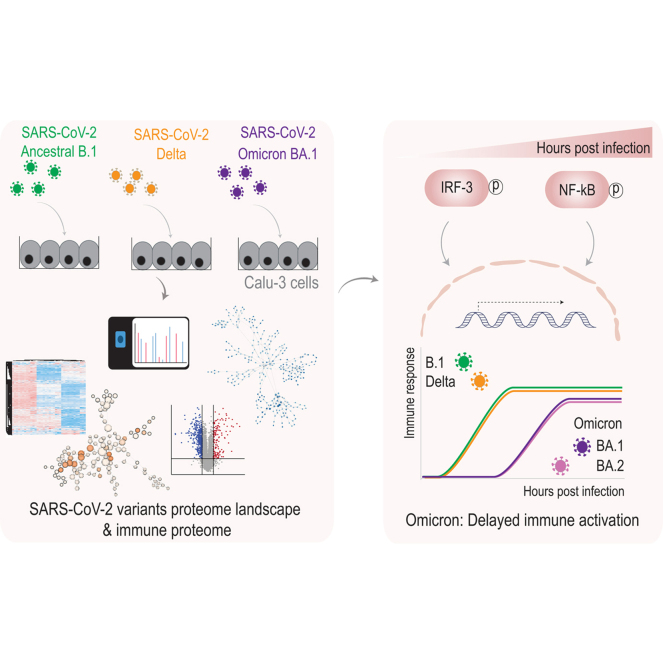
SARS-CoV-2 variants altered host proteome with delayed immune activation in Omicron-infected cells.

## In Brief

SARS-CoV-2 has mutated over the past years to numerous variants of concern (VOCs). Employing quantitative whole-cell proteomics, we elucidate host cell immune responses upon infection with ancestral B.1, VOCs Delta, and Omicron BA.1 strains. Molecular assays further illustrate reduced viral protein levels and delayed host immune pathway response upon infection with Omicron BA.1 when compared to B.1 and Delta. Overall, this study offers insights into host proteome profiles and distinct immune kinetics of ancestral B.1, Delta, and Omicron BA.1 strains.

In November 2019, a new coronavirus termed severe acute respiratory syndrome coronavirus 2 (SARS-CoV-2) was described in Wuhan (China), leading to the outbreak of the Coronavirus disease 2019 (Covid-19). The ancestral SARS-CoV-2 strain B.1 has rapidly mutated and numerous variants of concern (VOCs) have emerged, namely Alpha (B.1.1.7), Beta (B.1.351), Gamma (P.1), and Delta (B.1.617.2) (https://www.who.int/en/activities/tracking-SARS-CoV-2-variants. Accessed 18 September, 2022). As of November 2021, a new VOC named Omicron (BA.1 or B.1.1.529) was first described in South Africa, where it rapidly became the dominant variant. Several subvariants of Omicron have been described since then (e.g. BA.2, BA.4, or BA.5) (https://www.who.int/en/activities/tracking-SARS-CoV-2-variants. Accessed 18 September, 2022).

Omicron variants are of special interest, since they carry several mutations within the spike (S) protein, leading to an attenuated cell entry due to impaired TMPRSS2 cleavage (https://www.who.int/news/item/26-11-2021-classification-of-omicron-(b.1.1.529)-sars-cov-2-variant-of-concern. Accessed 11 September, 2022, https://outbreak.info/compare-lineages?pango=Omicron&pango=BA.2%2a%20%5BOmicron%20%28BA.2.X%29%5D&pango=B.1&pango=B.1.617.2&gene=S&gene=ORF1a&gene=ORF1b&gene=ORF3a&gene=ORF6&gene=ORF7a&gene=ORF7b&gene=ORF8&gene=ORF10&threshold=75&nthresh=1&sub=false&dark=false. Accessed 11 September, 2022). Above that, Omicron BA.1 was shown to possess immune escape (1, 2, 3), resulting in a higher risk of reinfection (4, 5). Consistent with these observations, there have been reports of reduced clinical efficacy of monoclonal antibodies used for antiviral treatment of high risk groups while small molecule drugs such as Remdesivir remained efficient (1, 6, 7). Furthermore, Omicron BA.1 was shown to be less pathogenic *in vivo* when compared to other VOCs (8, 9, 10), and less severe Covid-19 cases were reported in areas in which Omicron BA.1 was the dominating variant (8, 11, 12, 13, 14). Recent studies have confirmed the lower pathogenicity of BA.1, but less severe courses of Covid-19 are also due to the higher vaccination rate in the BA.1 wave than the Delta wave (15).

Recognition of RNA viruses, such as SARS-CoV-2, by intracellular pattern recognition receptors of host cells leads to activation of an innate immune response cascade (16). Consequently, downstream transcription factors, such as interferon regulator factor 3 (IRF-3) and nuclear factor κB (NF-κB), are phosphorylated and translocate into the nucleus, where they initiate the expression of type I and type III interferons (IFN) and proinflammatory cytokines, respectively (17, 18, 19, 20). However, the host cell IFN activation kinetic profile upon infection with different SARS-CoV-2 variants remains unclear.

In this study, we compared the host cell response upon infection with different SARS-CoV-2 variants to gain a better understanding of the host cell changes that may underlie varying pathogenicity and to elucidate host cell changes occurring during viral evolution. We compared the ancestral B.1 strain to the VOCs Delta and Omicron BA.1, which possess high pathogenicity and a high degree of immune escape, respectively. We carried out quantitative whole-cell proteomics using lung epithelial cells infected with different SARS-CoV-2 variants over time. Cells infected with Omicron BA.1 showed decreased viral proteome levels when compared to B.1 and Delta. Host immune response pathways were enriched upon infection with all variants. All SARS-CoV-2 variants showed nuclear translocation of IRF-3 and NF-κB as well as activation of IFN I and III responses. However, each variant possessed its individual kinetics in the induction of immune response, most pronounced with reduced inflammatory responses upon Omicron infection.

## Experimental Procedures

### Experimental Design and Statistic

Calu-3 lung epithelial cells were mock infected or infected with SARS-CoV-2 ancestral variant B.1, Delta, or Omicron BA.1 VOC at a multiplicity of infection (MOI) 1 and collected at 6, 12, and 24 hours post infection (hpi). Three biologically independent samples were processed for proteomics analysis. Protein samples were multiplexed into a 16-plex using tandem mass tags (TMTs), measured by quantitative mass spectrometry and raw data was analyzed using Proteome Discoverer (PD) 2.4 software (Thermo Fisher Scientific: https://thermo.flexnetoperations.com/control/thmo/product?plneID=820497). Proteomics data was analyzed further using PERSEUS (21) 1.6.15.0 software (https://maxquant.net/perseus/). Statistics determined using unpaired two-sided student’s t-test were additionally FDR corrected and values q < 0.05 were considered significant. Molecular assays were performed in three independent experiments with three replicates each. Analysis as well as graphical representation was conducted with Graph Pad Prism (version 9.3.1, GraphPad Software). Statistical significance was calculated by one or two-way ANOVA (ns, not significant, ∗*p* < 0.05, ∗∗*p* < 0.01, ∗∗∗*p* < 0.005, ∗∗∗∗*p* < 0.0001). Error bars are described in the figure legends.

### Cell Culture

Calu-3 cells were maintained in Dulbecco’s modified Eagle’s medium (DMEM)-F12 supplemented with 10 % fetal calf serum, 100 IU/ml of penicillin, and 100 μg/ml of streptomycin. DMEM-F12 was purchased from Gibco and all other materials from Sigma-Aldrich. HEK-Blue IFN-α/β and HEK-Blue IFN-λ cells (Invivogen) were cultivated in DMEM supplemented with 10% inactivated FCS, 50 U/ml penicillin, 50 μg/ml streptomycin, and 100 μg/ml Normocin (Invivogen). Selection antibiotics for the respective cell lines were as followed: IFN-α/β: 30 μg/ml blasticidin and 100 μg/ml Zeocin; IFN-λ: 10 μg/ml Blasticidin, 1 μg/ml Puromycin and 100 μg/ml Zeocin. Selective antibiotics were purchased from Invivogen. All cell lines were grown at 37 °C and 5 % carbon dioxide and tested for *mycoplasma* contamination on a regular basis.

### Virus Variants and Propagation

SARS-CoV-2 virus strains were propagated as described elsewhere (22). In brief, SARS-CoV-2 was grown on Caco-2 cells, and the Tissue Culture Infection Dose50 (TCID50) was calculated according to Spearman and Karber by titration of supernatants on 96-well plates of confluent Caco-2 (23, 24). Virus stocks were stored at −80 °C. The variants used in this study were as followed: B.1 (FFM7/2020; MT358643) (22), Delta B.1.617.2 (FFM-IND8424/2020; MZ31514) (25), Omicron BA.1/B.1.1.529a (2021; EPI_ISL_6959868), and Omicron BA.2/B.1.1.529 b (2022; EPI_ISL_6959871) (1).

### Virus Infection

For infection assays, cells were washed with PBS and maintained in medium with reduced FCS concentration (1 %). Cells were infected with SARS-CoV-2 at indicated MOI for 2 h and supplied with fresh medium after the incubation period. Virus containing samples were inactivated using validated protocols (26). All experiments with viable SARS-CoV-2 were performed under Biosafety Level-3.

### Immunofluorescence

Cells were fixed with 3 % paraformaldehyde for 30 min and subsequently permeabilized with 0.5 % Triton-X for 30 min. After blocking for 1 h with 1 % bovine serum albumin, primary antibody was diluted in 0.5 % bovine serum albumin and the solution incubated overnight at 4 °C. The secondary antibody mixed with DAPI (0.02 mg/ml) was incubated for 1 h at room temperature (RT). Primary antibodies used were as followed: mouse anti SARS-CoV-2 Spike (GeneTex #GTX632604 1:1000), rabbit anti SARS-CoV-2 Spike (Sino Biological #40150-R007 1:1000), rabbit anti IRF-3 (Cell Signaling #4302 1:1000), rabbit anti NF-κB (Cell Signaling #8242 1:1000). Secondary antibodies used were as followed: goat anti mouse Alexa 488 (Invitrogen #A11001 1:1000), goat anti rabbit Alexa 488 (Invitrogen #A11008 1:1000), goat anti rabbit Alexa 647 (Invitrogen, #A21244 1:1000). Images were acquired using the Operetta CLS High Content Analysis System (PerkinElmer) followed by image analysis with Harmony (PerkinElmer).

### Quantification of Intracellular Virus RNA

Infected cells were lysed with RLT buffer (QIAGEN), and RNA was isolated using the RNeasy 96 QIAcube HT Kit (QIAGEN) according to the manufacturer’s instructions. Quantitative PCR (qPCR) was performed with the Luna Universal One-Step RT-qPCR Kit (New England Biolabs) using a CFX96 Real-Time System (Bio-Rad). Primers for GAPDH were as followed: GAPDH_fw (TGCACCACCAACTGCTTA), GAPDH_rev (GGATGCAGGGATGATGTTC). Primers targeting the RNA-dependent RNA polymerase (RdRp) were adapted from the WHO: RdRP_SARSr-F2 (GTGARATGGTCATGTGTGGCGG) and RdRP_SARSr-R1 (CARATGTTAAASACACTATTAGCATA).

### Proteomics

For whole-cell proteomics, Calu-3 cells were mock infected (time point 0) or infected with SARS-CoV-2 variants (MOI 1) B.1, Delta, Omicron BA.1 and collected at 6, 12, 24 hpi (supplemental Tables S1 and S2). Samples were collected in lysis buffer (100 mM Tris, 2 % SDS, 10 mM TCEP, 40 mM 2-CAA) and sample lysates for total assays were performed as described in (27, 28). Briefly, sample lysates were methanol/chloroform precipitated and resuspended in buffer containing 8 M Urea and 100 mM Tris (PH.8). Protein concentration was determined by Bradford assay and 300 μg of protein per sample was used for digestion after dilution to 1M Urea and 100 mM Tris. Samples were digested with 1:50 wt/wt LysC and 1:100 wt/wt Trypsin overnight at 37 °C. Digested samples were acidified with TFA and peptides were purified using Waters Oasis Prime HLB 30 mg columns according to manufacturer`s instructions. Dried peptide samples were resuspended in TMT labeling buffer containing 200 mM EPSS and 10 % acetonitrile and incubated at 37 °C for 10 min. Peptide concentration was determined by μBCA and 100 μg of peptides per sample were used for TMT labeling (TMTpro-16) by one-hour incubation at RT using a 1:2.5 peptide/TMT-ratio. The reaction was quenched by the addition of 1:10 (vol) 5 % hydroxylamine solution at RT for 15 min. TMT labeling quality was verified by mixing equimolar ratios of each TMT channel followed by single injection measurement by LC-MS/MS. Samples were pooled acidified using 20 % TFA and purified using SepPak (Waters Oasis Prime HLB 30 mg columns). For whole-cell proteome, pooled peptides were used for High pH Reverse phase fractionation by Dionex Ultimate 3000 analytical HPLC (27, 28). The eluted peptides were collected for 96 fractions and cross concatenated into 24 fractions and dried for processing. Liquid chromatography and mass spectrometry were performed as described previously in (27, 28).

Mass spectrometry raw data analysis was performed using Proteome Discoverer (PD) 2.4 software (Thermo Fisher Scientific). Default settings were used for the selection of spectra. SequestHT node was opted for database searches against trypsin-digested *Homo sapiens* reference proteome (Taxonomy ID 9606) downloaded from UniProt (12-March-2020; “One Sequence Per Gene”, 20,531 entries) and SARS-CoV-2 (UniProt pre-release, 10-February-2020, Taxonomy ID 2697049; 14 entries). Precursor mass tolerance of 7 ppm and a fragment mass tolerance of 0.5 Da was set in the database search. Static modifications were set as TMTpro at the N terminus and carbamidomethyl at cysteine residues. The following dynamic modifications were taken into account: Oxidation (M) and Acetyl (Protein N terminus). False discovery rates were controlled using Percolator <0.01 FDR at peptide and <0.05 FDR at protein level. For whole-cell proteomics quantification, all peptide spectrum matches were summed intensity normalized, followed by internal reference scaling (29) normalization. Further data analysis was performed using PERSEUS (21) 1.6.15.0 software. Significance was tested using unpaired two-sided student’s t-test and values were further FDR corrected. Values q < 0.05 were considered significant. Gene Ontology (GO) category enrichment analysis for proteomic dataset was performed using DAVID functional annotational tool (30) (DAVID Bioinformatics Resources 6.8) and further analysis was performed using Enrichment map v3.2.1, OmicsVisualize v1.3.0, and STRING v1.5.1 application on Cytoscape (31) v 3.7.1.

### Immunoblot Analysis

Confluent Calu-3 cells in 6-well plates were lysed using Triton-X lysis buffer supplemented with protease and phosphatase inhibitors (Roche) and left on ice for 30 min. Samples were mixed with an equal volume of Laemmli buffer (Sigma) supplemented with 5 % ß-mercaptoethanol. Subsequently, samples were incubated at 95 °C for 15 min and stored at −20 °C. For examining IFN pathway, proteins were separated by SDS-PAGE followed by blotting on polyvinylidene difluoride membranes. Upon blocking in TBST with 5% w/v nonfat dry milk for 1 h, membrane was incubated with primary antibody overnight at 4 °C followed by secondary antibody incubation for 1 h at RT. Antibodies used were as followed: rabbit anti IRF-3 (Cell Signaling #4302 1:1000), rabbit anti IRF-3 (S386) (Cell Signaling #37829 1:1000), goat anti mouse HRP (Jackson Immunoresearch #115-035-062 1:10,000), goat anti rabbit HRP (Merck #A6154 1:10,000). Images were acquired with the ChemiDoc MP imaging system (Bio-Rad) and quantification was conducted with imageJ (1.53 t). For assessing NF-κB pathway, proteins were separated with SDS-PAGE (Invitrogen Novex system) and transferred to nitrocellulose membrane using Mini Trans-Blot (Bio-Rad). Membranes were blocked for 1 h using Intercept blocking buffer (LI-COR) and probed overnight at 4 °C with the following primary antibodies: NF-κB p65 (C-20) (Santa Cruz #372, 1:1500, rabbit), Phospho- NF-κB p65 (Ser536) (Cell Signaling #3031,1:1000, rabbit), IkBa (c-21) (Santa Cruz #371, 1:1000, rabbit), and Actin (Santa Cruz #69879, 1:2000, mouse). Appropriate secondary antibodies were used for imaging with Odyssey DLx (LI-COR) and quantitation was performed using Image Studio lite v5.2.

### Detection of Bioactive IFNαß and IFNλ

HEK-Blue IFN-α/β and HEK-Blue IFN-λ reporter cells were used to examine the release of interferons from SARS-CoV-2–infected Calu-3 cells according to the manufacturer’s instructions. Therefore, supernatants of infected Calu-3 cells were stored upon analysis at −80 °C. For the assay, HEK-Blue cells were washed twice with PBS, resuspended in fresh test medium (growth medium without selection antibiotics), and adjusted to a cell number of 280.000 cells/ml. Twenty microliters of the supernatant of infected Calu-3 cells were added to a 96-well plate followed by 180 μl of HEK blue cell suspension and incubated at 37 °C and 5 % carbon dioxide. After 24 h, 20 μl supernatant of HEK blue reporter cells were transferred to a 96-well plate followed by 180 μl of QUANTI-Blue solution (Invivogen) and incubated at 37 °C for 30 to 120 min. Reporter activity (SEAP levels) was determined by absorbance at 620 nm using a spectrometer.

## Results

### Replication Kinetics of SARS-CoV-2 Variants in Calu-3 Lung Cells

**Fig. 1:**
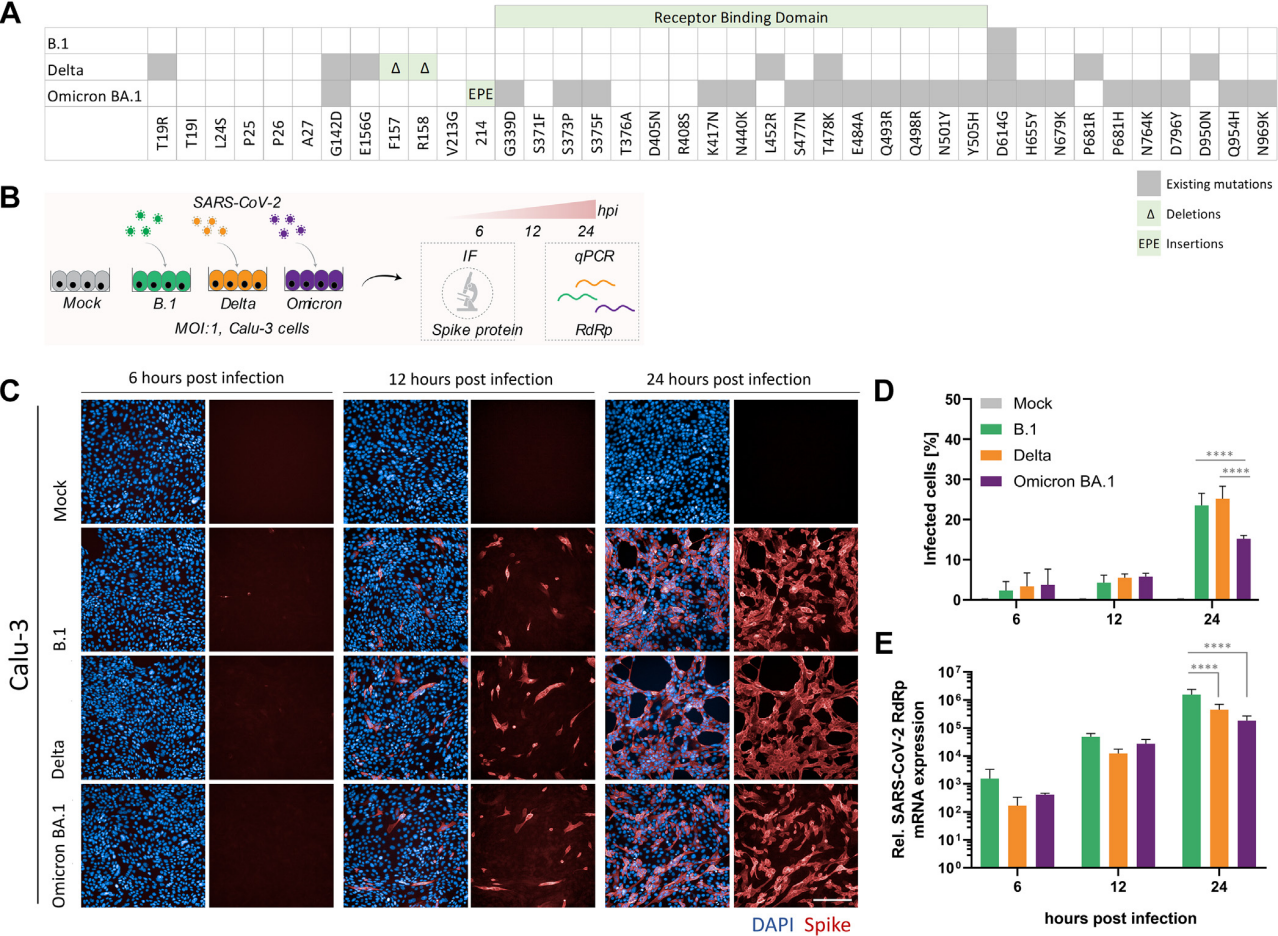
**Viral kinetics of SARS-CoV-2 variants in Calu-3 cells**. *A*, mutational landscape of SARS-CoV-2 variants within the S protein (*gray* boxes: existing mutations; *triangles*: deletions; letters: insertions; source: https://outbreak.info/compare-lineages?pango=Omicron&pango=BA.2%2a%20%5BOmicron%20%28BA.2.X%29%5D&pango=B.1&pango=B.1.617.2&gene=S&gene=ORF1a&gene=ORF1b&gene=ORF3a&gene=ORF6&gene=ORF7a&gene=ORF7b&gene=ORF8&gene=ORF10&threshold=75&nthresh=1&sub=false&dark=false. Accessed 11 September, 2022. *B*, experimental scheme. *C*, representative immunofluorescence images of Calu-3 cells infected with SARS-CoV-2 variants at MOI 1 (*blue*: DAPI; *red*: S protein; scale bar represents 200 μm). *D*, quantification of SARS-CoV-2–infected cells based on immunofluorescence images using Harmony software (PerkinElmer). *E*, SARS-CoV-2 RNA kinetics measured with qPCR of the RdRp. Statistics of (*D* and *E*) were determined by two-way ANOVA (n = 3 biologically independent replicates), bars represent the mean and error bar show ±SD. ∗∗∗∗*p* < 0.0001. MOI, multiplicity of infection; qPCR, quantitative PCR; RdRp, RNA-dependent RNA polymerase; SARS-CoV-2, severe acute respiratory syndrome coronavirus 2.

The SARS-CoV-2 VOC Omicron BA.1 carries several mutations within the Spike (S) protein resulting in impaired cell entry (Fig. 1*A*). To identify resulting changes in cellular replication, we determined BA.1 replication kinetics in comparison to the ancestral strain B.1 and the VOC Delta. The lung cell line Calu-3 was infected at an MOI of 1 and the viral load determined at different time points by immunofluorescence staining and qPCR analysis (Fig. 1*B*). Notably, the most severe cytopathic effect (CPE) was observed in cells infected with the Delta variant followed by B.1 with CPE scarcely observable in Omicron BA.1–infected cells (Fig. 1*C*). At 24 hpi, cells infected with B.1 and Delta showed a significantly higher number of infected cells than Omicron BA.1 based on S protein staining (Fig. 1*D*). Moreover, intracellular viral RNA levels were significantly higher at 24 hpi in B.1 infected cells than in cells infected with Omicron BA.1 and Delta variants, with Omicron BA.1 displaying the lowest among all SARS-CoV-2 variants (Fig. 1*E*). Overall, Omicron BA.1 showed slower replication kinetics than B.1 or Delta.

### Host Cell Proteome Profile Remodeling After Infection With SARS-CoV-2 Variants

**Fig. 2:**
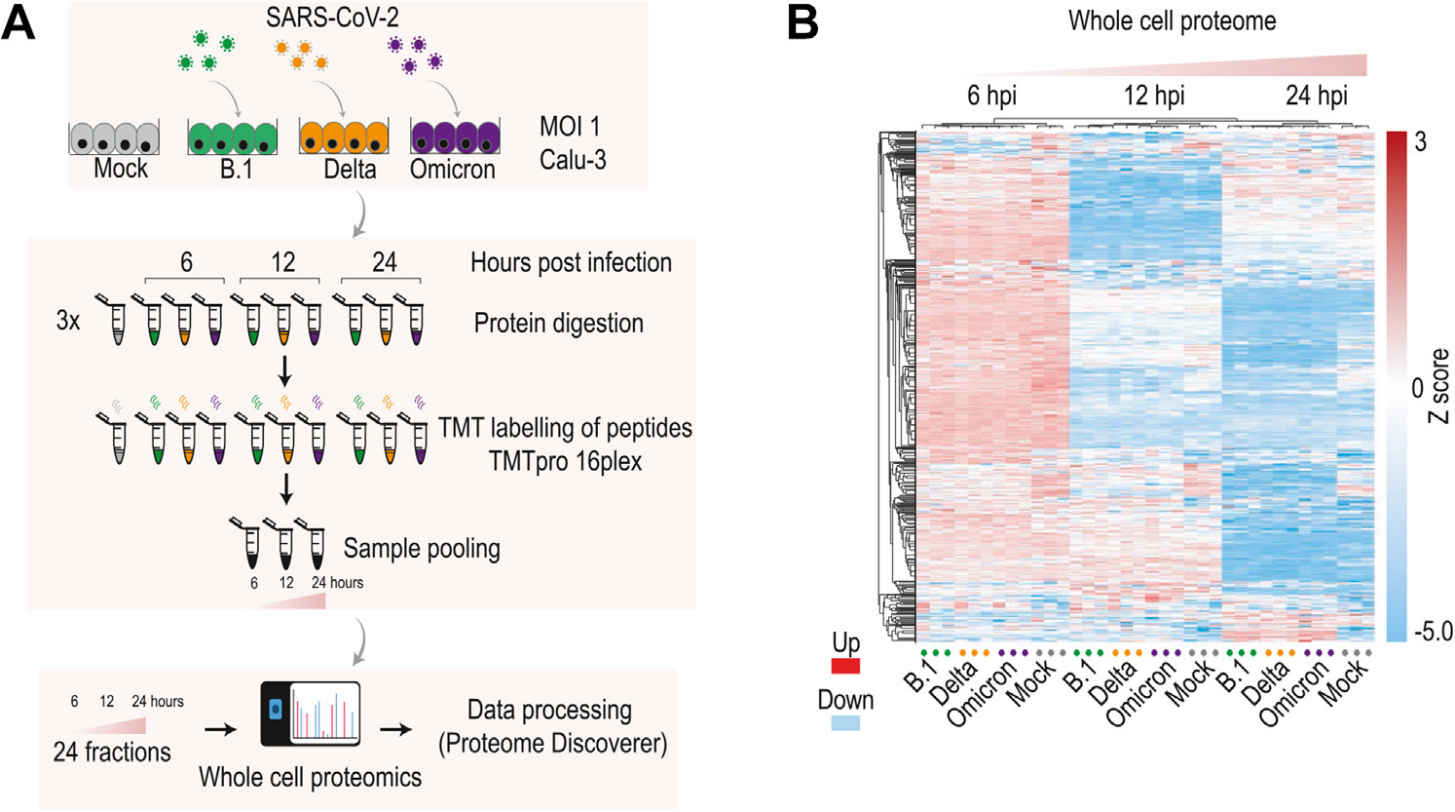
**Global host proteome profile upon SARS-CoV-2 variant infection.***A*, schematic representation of experimental workflow: Calu-3 cells were mock infected or infected (MOI 1) with SARS-CoV-2 variants B.1, Delta, or Omicron BA.1 and collected at 6, 12, and 24 hpi (n = 3 biologically independent replicates). *B*, heat map represents Z-scores of differentially expressed peptides identified in total proteomic analysis in sample groups and hpi as indicated (increased: *red* and decreased: *blue*). hpi, hours post infectio; MOI, multiplicity of infection; SARS-CoV-2, severe acute respiratory syndrome coronavirus 2.

To decipher alterations in the host cell proteome and cellular signaling pathways upon infection with different SARS-CoV-2 variants that may drive the observed replication differences, we performed unbiased quantitative proteomics analyses of infected cells. Calu-3 cells mock infected or infected with SARS-CoV-2 variants B.1, Delta, or Omicron BA.1 (MOI 1) were collected at 6, 12, and 24 hpi (Fig. 2*A*). Protein samples were multiplexed into a 16-plex using TMTs and analyzed by quantitative mass spectrometry. Across all conditions, 8023 differentially expressed proteins were quantified (Fig. 2*B*). Principle component analysis displayed separation of all SARS-CoV-2–infected samples from mock-infected samples already at 6 hpi. Replicates of SARS-CoV-2 variants B.1-, Delta-, and Omicron BA.1–infected samples grouped into distinct clusters starting from 12 hpi. Variants formed highly distinctive clusters at 24 hpi (Figs. 2*B* and S1, *A*–*C*). Overall, the proteomic data revealed alterations in global host cell proteome upon SARS-CoV-2 variant infection.

### Reduced Viral Proteome Expression for Omicron BA.1

**Fig. 3:**
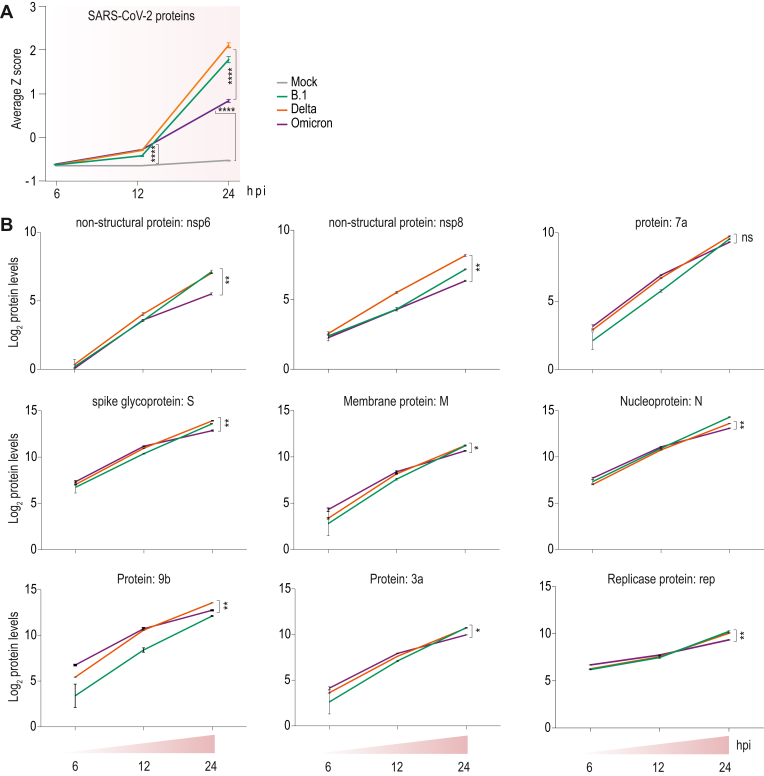
**Viral proteome kinetics of SARS-CoV-2 variants.***A*, average Z-score of viral proteins detected upon infection with SARS-CoV-2 variants B.1, Delta, Omicron BA.1, or mock control in the total proteome analysis at 6, 12, and 24 hpi. *B*, protein abundance plotted as log_2_-transformed values of detected SARS-CoV-2 viral proteins, significance determined between Omicron BA.1 vs Delta (supplemental Table S3). Statistics were determined as described in proteomics method section, FDR *q*-value <0.05 was considered significant (n = 3 biologically independent replicates), error bar show ±s.e.m. ∗ q < 0.05, ∗∗ q < 0.02, ∗∗∗∗ *p* < 0.0001 ns: not significant. hpi, hours post infection; SARS-CoV-2, severe acute respiratory syndrome coronavirus 2.

To gain a better understanding of infection kinetics across the different variants, we next monitored viral protein levels over time. We detected nine viral proteins across all variants. All increased over time, indicating productive infection. While all SARS-CoV-2 variants showed similar expression patterns at 12 hpi, Omicron BA.1 displayed significant lower levels of most viral proteins at 24 hpi than B.1 and Delta infection (Fig. 3, *A* and *B* and supplemental Table S3). This observation was consistent with the decreased number of infected cells observed for Omicron BA.1 (Fig. 1). Thus, the viral proteome data suggests a lower replication rate of Omicron BA.1.

### Induction of Cellular Immune Response Pathways upon SARS-CoV-2 Variant Infection

**Fig. 4:**
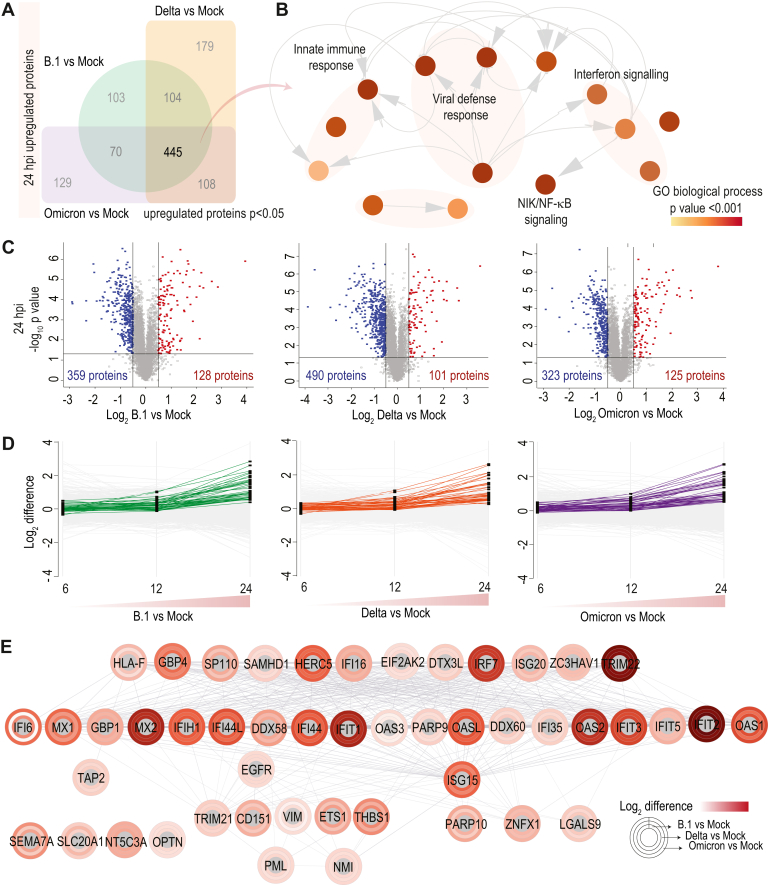
**Immune profile upon SARS-CoV-2 variant infection.***A*, Venn diagram represents overlap of significantly (*q*-value ≤0.05) upregulated proteins at 24 hpi in Calu-3 cells infected with SARS-CoV-2 variants B.1, Delta, or Omicron BA.1 in comparison to mock. *B*, functional GO enrichment analysis (*p*-value ≤0.001), category biological process (BP) for overlapping upregulated proteins at 24 hpi. *C*, volcano plots depict significantly upregulated (*red*) and downregulated (*blue*) peptides (*q*-value ≤0.05, −0.5 ≤ log2 difference ≥0.5) from total proteomic analysis in comparisons: B.1 vs mock, Delta vs mock, and Omicron BA.1 vs mock at 24 hpi. *D*, profile plot shows log_2_ difference of proteins under GO BP from (*A*) with (*q*-value ≤0.05, log2 difference ≥0.5 at 24 hpi) in comparisons: B.1 vs mock, Delta vs mock, and Omicron BA.1 vs mock at time points as indicated. *E*, STRING enrichment analysis network of proteins from (*D*) with expression pattern representing log_2_ difference at 24 hpi in comparisons as indicated. BP, biological process; GO, gene ontology; hpi, hours post infection; SARS-CoV-2, severe acute respiratory syndrome coronavirus 2.

To attain insight into variant-specific host cell changes, we next examined host cellular proteins that significantly changed across SARS-CoV-2 variants in comparison to mock-infected cells (supplemental Table S4). We observed activation of immune response and suppression of processes involved in cell cycle, DNA repair, and nucleic acid metabolism at 12 hpi (supplemental Fig. S2, *A*–*D*). Similarly, cellular processes such as cell cycle, DNA repair, and replication were reduced at 24 hpi (supplemental Fig. S2*E*). GO analyses revealed the enrichment of several biological processes that mediate host cellular viral and immune response signaling pathways, especially the induction of proteins representing immune pathways such as IFN and NF-κB signaling, upon infection of SARS-CoV-2 B.1, Delta, and Omicron BA.1 variants (Fig. 4, *A* and *B*).

To better understand the temporal trajectory of host antiviral pathway proteins, we compared significantly changing immune pathway proteins that were highly enriched in SARS-CoV-2 variants at 24 hpi (*q*-value ≤0.05, SARS-CoV-2 variants vs mock, log_2_ difference ≥0.5) (Fig. 4, *B* and *C*). Antiviral pathway proteins showed similar patterns of activation between variants and across time points, with the highest abundance observed at 24 hpi (Fig. 4, *D* and *E*), the time at which the highest viral protein abundances were observed (Figs. 1 and 3). This suggests that the host cell rewires itself in response to SARS-CoV-2 infection and responds by increasing immune response proteins, emphasizing the eminent role of antiviral signaling pathways in shielding against multiple SARS-CoV-2 variants (32, 33).

### Comparing the Host Proteome Landscapes Upon Omicron BA.1 or Delta Infection

**Fig. 5:**
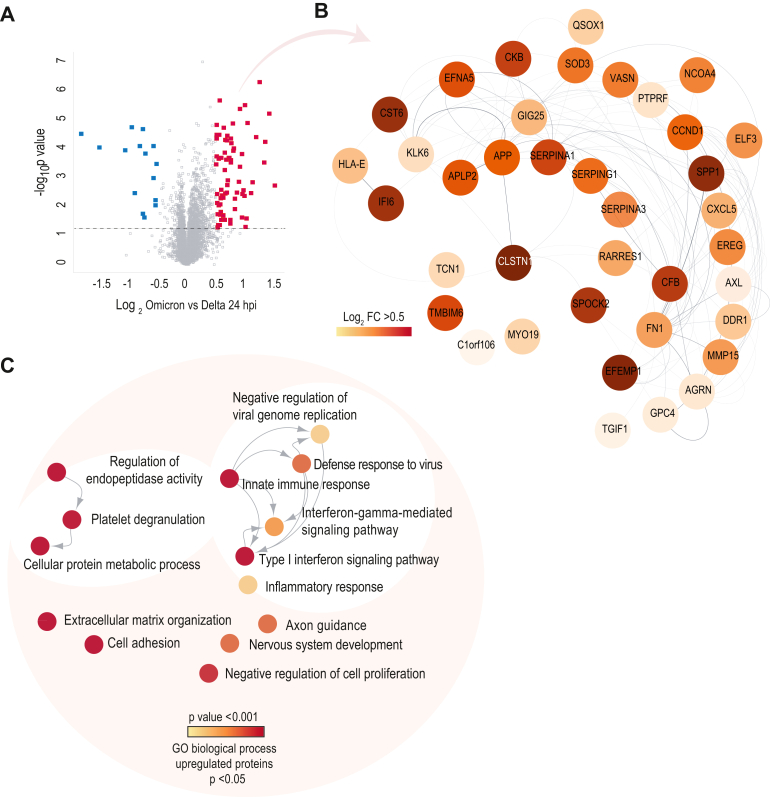
**Proteome landscape of OmicronBA.1- orDelta–infected cells.***A*, volcano plot depicts significantly increased (*red*) and decreased (*blue*) proteins (*q*-value ≤0.05, −0.5 ≤ log_2_ difference ≥0.5) from total proteome analysis in comparison to Omicron BA.1 vs Delta at 24 hpi. *B*, STRING enrichment analysis of protein network for significantly (*q*-value ≤0.05, log_2_ difference ≥0.5) increased host proteins in comparison to Omicron BA.1 vs Delta from infected cells at 24 hpi. *C*, functional GO enrichment analysis (*p*-value <0.001), category biological process for significantly (*q*-value ≤0.05) increased (*red*) proteins. hpi, hours post infection; GO, gene ontology.

Despite the rapid increase in SARS-CoV-2 proteins post infection, few host proteins changed in abundance between SARS-CoV-2 variant infected cells at 6 and 12 hpi (supplemental Fig. S3*A* and supplemental Table S5). However, major differences in host proteome profile were observed between Omicron BA.1– and Delta-infected cells at 24 hpi (supplemental Fig. S3*A* and supplemental Table S5). Comparing SARS-CoV-2 Omicron BA.1 and Delta variants identified 15 and 104 proteins that were significantly decreased or increased, respectively (Figs. 5, *A* and *B* and S3*B*). We wondered what host cellular processes drives the variations observed between Omicron- and Delta-infected cells. GO enrichment analysis of host proteins that were significantly increased upon infection between Omicron BA.1 and Delta revealed enrichment of processes regulating endopeptidase activity, nervous system development, axon guidance, cell adhesion, and proliferation (Fig. 5, *B* and *C*). Interestingly, multiple protease inhibitors such as SERPINA1, SERPINA3, and SERPING1 of SERPIN family of proteins known to regulate inflammation and endopeptidase activity were among the highly enriched proteins (Fig. 5*B*).

Moreover, cellular viral and immune response pathways including IFN signaling were induced (Fig. 5*C*). Notably, although we observed similar activation of immune antiviral proteins among SARS-CoV-2 variants, a minor subset showed significant difference in expression upon Omicron BA.1 infection compared to Delta (Fig. 4*E*). These proteins included immune and viral defense response proteins, such as TRIM25, PARP10, and MX1. Moreover, proteins regulating IFN pathway such as DDX58, IRF6, OAS2, OAS3, and STAT2, along with interferon-induced proteins IFIT2 and IFI6, showed increased expression (supplemental Fig. S3*C*). Interestingly, the increase of IFN pathway proteins points to the antiviral activity of the host cell to counteract viral replication. These data suggest that Omicron BA.1 infection alters host proteome and immune response profiles in a manner distinct from Delta.

### Interferon and NF-κB Signaling Induced by Infection With SARS-CoV-2 Variants

Since the proteome analysis revealed evident increases of host cell immune signaling proteins corresponding to antiviral pathways including IFN and NF-κB signaling, we analyzed phosphorylation patterns of IRF-3 and NF-κB using immunoblots. IRF-3 is known to induce the expression of type I and III interferons and NF-κB to induce the expression of proinflammatory cytokines (19, 20). We also included the formerly dominating variant BA.2, which was not circulating at the time of proteome analyses. Interestingly, viral levels of Omicron BA.2 were even lower than those of Omicron BA.1 (supplemental Fig. S4).

**Fig. 6:**
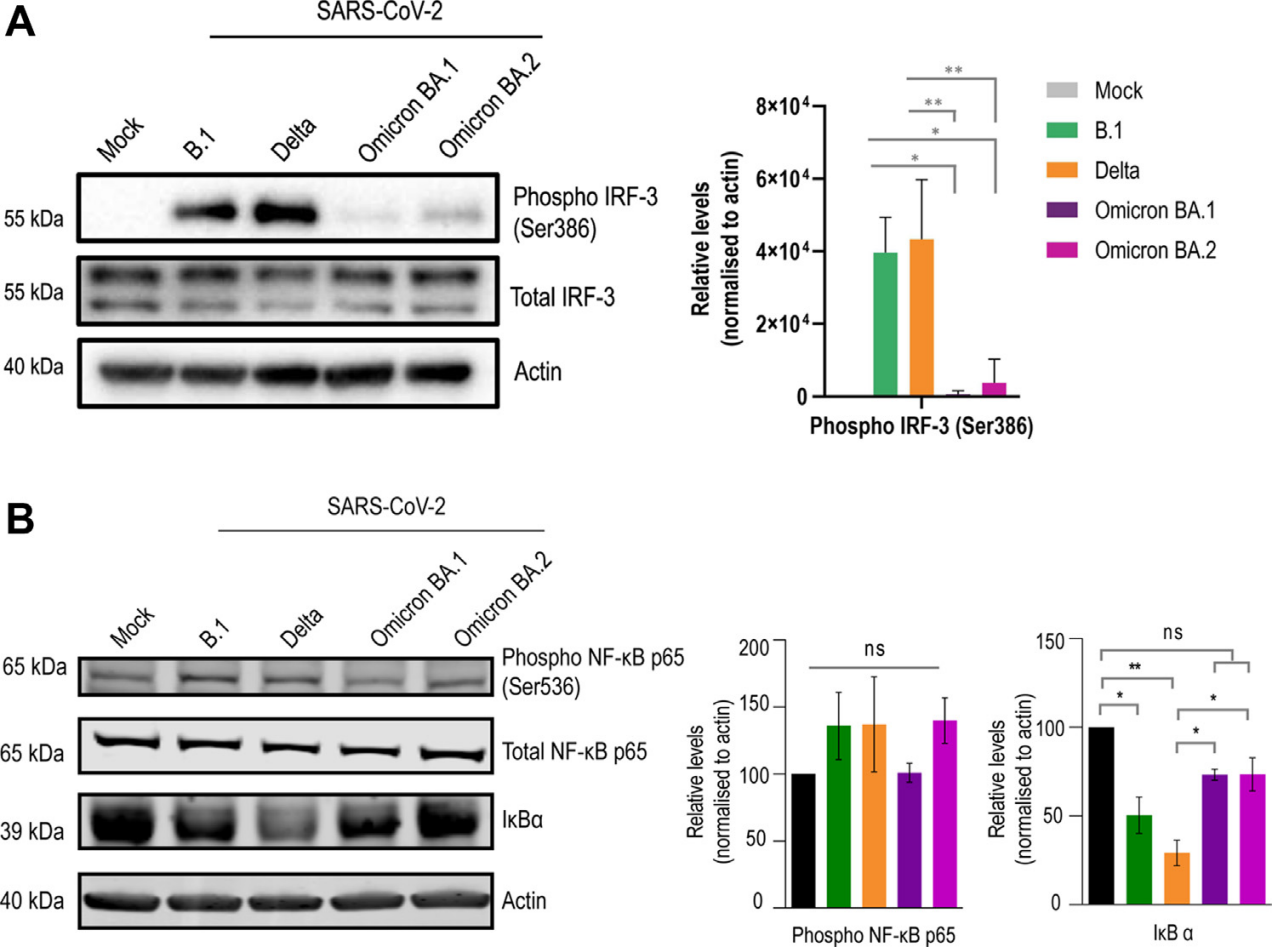
**Phosphorylation of IRF-3 and NF-kB upon infection with SARS-CoV-2 variants.** Calu-3 cells were infected with SARS-CoV-2 variants at an MOI of 1 for 24 h. Representative immunoblot and quantification of (*A*) IRF-3 and pIRF-3 (S386), (*B*) Total NF-κB, NF-κB (p65) phosphorylation and Iκb alpha. Statistics were determined by two-way ANOVA (n = 3 biologically independent replicates), bars represent the mean and error bar shows ±SD/s.e.m, ∗*p* < 0.5, ∗∗*p* < 0.01, ns, not significant. IRF, interferon regulator factor; MOI, multiplicity of infection; NF-κB, nuclear factor κB; SARS-CoV-2, severe acute respiratory syndrome coronavirus 2.

For the analysis, Calu-3 cells were infected with an MOI of 1 and lysed after 24 hpi, as the proteome analysis revealed major differences in host immune response at this time point. Strikingly, immunoblot assay revealed significant differences between Omicron BA.1 and BA.2 and the other variants. High levels of phosphorylated IRF-3 were detected in B.1- and Delta-infected cells, while cells infected with Omicron BA.1 or Omicron BA.2 showed no or little IRF-3 phosphorylation (Fig. 6*A*). Total IRF-3 levels did not change. Consistently, a significant reduction of the NF-κB inhibitor IκB alpha was observed in B.1- and Delta-infected cells, and a mild induction of NF-κB phosphorylation (p65) was observed in B.1, Delta, and Omicron BA.2 (Fig. 6*B*). Altogether, this data indicates that the activation of specific antiviral signaling pathways (*i.e.* phosphorylation of IRF-3 and IκB alpha stabilization) was observed predominantly in cells infected with the variants B.1 and Delta. This observation suggests that infection with Omicron BA.1 and BA.2 elicits a divergent immune response when compared to other SARS-CoV-2 variants.

### Nuclear Translocation of Key Transcription Factors and IFN Release Upon SARS-CoV-2 Variant Infection

**Fig. 7:**
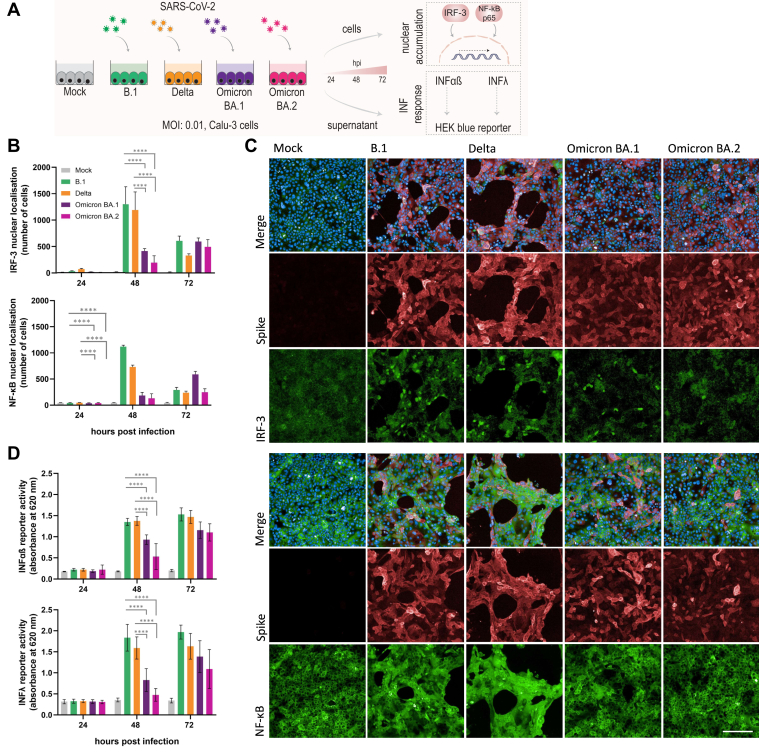
**Kinetics of innate immune response induction upon infection with different SARS-CoV-2 variants**. *A*, experimental scheme. Calu-3 cells were infected with different SARS-CoV-2 variants at an MOI of 0.01 for 24 h, 48 h, and 72 h and immune response monitored. *B*, nuclear translocation of IRF-3 and NF-κB was quantified with immunofluorescence using the Harmony software (PerkinElmer). *C*, representative immunofluorescence images of IRF-3– and NF-κB–stained cells at 48 hpi (*blue*: DAPI, *green*: IRF-3 or NF-κB, *red*: spike, scale bar represents 200 μm). *D*, interferon release was measured with HEK blue reporter cells for IFNαß and IFNλ release. Statistics of (*B* and *D*) were determined by two-way ANOVA (n = 3 biologically independent replicates), bars represent the mean and error bar shows ±SD. ∗∗∗∗*p* < 0.0001. hpi, hours post infection; IRF, interferon regulator factor; MOI, multiplicity of infection; NF-κB, nuclear factor κB; SARS-CoV-2, severe acute respiratory syndrome coronavirus 2.

Phosphorylated IRF-3 and NF-κB accumulate in the nucleus, where they act as transcription factors inducing the expression of type I and III interferons and proinflammatory cytokines, respectively (17, 18, 19, 20). Thus, we next examined nuclear localization of IRF-3 and NF-κB by quantitative fluorescence microscopy (Fig. 7*A*). We used an lower MOI of 0.01, in order to gain a higher temporal resolution. Notably, due to the strong CPE induction of B.1 and Delta, we did not use the time point 72 hpi for further evaluation, as less than 50 % and 25 % cells remained in B.1- and Delta-infected samples, respectively (supplemental Fig. S5*A*). Extensive nuclear translocation was first observed at 48 hpi, whereby cells infected with Omicron BA.1 and BA.2 variants had a significantly lower number of cells with IRF-3 and NF-κB nuclear localization in comparison to B.1- and Delta-infected cells (Fig. 7, *B* and *C*). Nuclear localization of IRF-3 and NF-κB increased further at 72 hpi in Omicron BA.1- and BA.2-infected cells (Supplemental Fig. S5B). However, it remained below the maximal activation observed for B.1 and Delta (supplemental Fig. S5*C*). Together, we observed more pronounced antiviral signaling *via* IRF-3 and NF-κB in B.1- and Delta-infected lung cells compared to Omicron BA.1 and BA.2 variants.

As IRF-3 translocation leads to the expression of type I and III interferons (IFNαß and IFNλ) (20), we next analyzed the cells for interferon release. In order to do so, supernatants of infected cells were analyzed with HEK blue reporter cells for bioactive IFNαß and IFNλ (Fig. 7*A*). In line with the lack of IRF-3 and NF-κB translocation, no interferon induction was observed at 24 hpi. However, high levels of IFNαß and IFNλ release were detected from cells infected with B.1 and Delta variants at 48 hpi, while levels were significantly lower upon Omicron BA.1 and BA.2 infections (Figs. 7*D* and S5*D*). Moreover, taking into account different viral kinetics of the variants (Fig. 1), interferon induction of B.1 and Delta at 48 hpi was compared with interferon induction of Omicron variants at 72 hpi, revealing differences of either low significance or no significance at all (supplemental Fig. S5*E*).

In summary, by different orthogonal assays, we observed a deferred immune response upon Omicron infection when compared to the other variants examined in this study.

## Discussion

In comparison to other SARS-CoV-2 VOCs, Omicron variants are particularly outstanding for their immune escape and altered pathogenicity (1, 2, 3, 8, 9). An unbiased assessment of the host cellular immune response upon infection of an Omicron variant was therefore essential. In this study, we performed unbiased proteomics combined with molecular assays to compare the host immune response of Omicron BA.1 to the highly pathogenic VOC Delta and ancestral B.1 to get a deeper understanding of variations in immune response of the host cell.

Omicron BA.1 was shown to possess superior replication kinetics in human nasal and bronchial tissue, but attenuated replication kinetics were reported in human lung tissue compared to other SARS-CoV-2 variants (34, 35). Notably, although the viral kinetics of Omicron variants were partly different, the replication in lung tissue was impaired in all *in vivo* models (8, 9, 10, 36, 37). The reported lower pathogenicity and increased transmissibility of the Omicron BA.1 variant possibly goes along with a shifted cell tropism. A possible explanation of this shift in cell tropism is inefficient TMPRSS2-mediated cleavage of the S protein of Omicron variants, which results in cell entry dependency on endosomal uptake as well as less fusogenicity (9, 38). However, replication kinetics were comparable to SARS-CoV-2 Delta in cells with low endogenous TMPRSS2 expression, like HeLa or 293T cells (36, 38). This explains our findings of attenuated infection kinetics as well as less severe CPE formation of TMPRSS2-expressing lung epithelial Calu-3 cells infected with Omicron BA.1 and BA.2 variants. Our proteomics analysis further validated impaired replication kinetics on a variety of viral proteins. Interestingly, decreased infectious viral loads were observed in Covid-19 patients infected with the Omicron BA.1 variant (39). Considering the reported enhanced infectivity of Omicron BA.1 (8, 11, 12, 13, 14), it is feasible to speculate that the impaired replication kinetics of the Omicron BA.1 variant in lung tissue are a strategic trade-off for its immune escape.

Our unbiased proteomics analysis reveals the crucial rewiring of host cellular immune proteome landscape upon infection of multiple SARS-CoV-2 variants. Upon viral infection, the host cell activates an immune and antiviral cascade as a pivotal line of defense (20). Similarly, host proteome upon infection of all SARS-CoV-2 variants examined in this study showed an enrichment of immune and antiviral response proteins including factors representing signaling pathways such as IFN and NF-κB (17, 18, 19, 20). Notably, in this study, a small subset of proteins corresponding to IFN signaling pathways was significantly increased in Omicron BA.1–infected samples. In the same line, a previous study investigating the proteome of serum samples from Omicron BA.1-infected patients revealed comparable host immune responses of SARS-CoV-2 Omicron BA.1– and Delta-infected patients. However, inflammation-associated pathways were enriched in Omicron BA.1–infected patients (40).

Activation of key immune signaling pathways identified from our proteome analysis are mediated by posttranscriptional modifications (17, 19). Therefore, examining the phosphorylation pattern and nuclear translocation post infection of key transcription factors IRF-3 and NF-κB revealed Omicron BA.1 and BA.2 variants to induce a delayed host immune response. In line with this observation, Omicron BA.1 infection was shown to be accompanied by less severe lung inflammation as well as the downregulation of proinflammatory cytokines *in vivo* compared to infection with other variants, possibly due to its impaired replication in lung tissue (8, 9, 36, 37)). In addition, Omicron BA.1–infected patients showed a low systemic inflammatory response compared to patients infected with previous SARS-CoV-2 variants (41). Mechanistically, the direct comparison of the B.1 S protein with the BA.1 S protein revealed that the BA.1 S protein induced less activation of immune modulators, such as NF-κB (42).

Notably, SARS-CoV-2 was shown to have numerous strategies to weaken the host cell immune response. Most prominently, SARS-CoV-2 ORF6 and the proteases PLpro and 3CLpro were shown to inhibit IRF-3–induced type I interferon expression, either by preventing its nuclear localization or by cleavage of IRF-3 dimers (43, 44, 45, 46). Strikingly, several mutations within these proteins were observed in Omicron BA.1 and BA.2 (https://outbreak.info/compare-lineages?pango=Omicron&pango=BA.2%2a%20%5BOmicron%20%28BA.2.X%29%5D&pango=B.1&pango=B.1.617.2&gene=S&gene=ORF1a&gene=ORF1b&gene=ORF3a&gene=ORF6&gene=ORF7a&gene=ORF7b&gene=ORF8&gene=ORF10&threshold=75&nthresh=1&sub=false&dark=false. Accessed 11 September, 2022). In addition, the Omicron BA.1 variant was shown to be highly sensitive to interferon treatment, which could be another explanation for the attenuated replication kinetics and lower pathogenicity of Omicron variants than B.1 and Delta (7, 47).

In summary, we show that host immune proteins are commonly enriched upon infection of all SARS-CoV-2 variants examined in this study. However, the kinetics of antiviral signaling at the level of post translational modification, cellular localization, and the resulting interferon response are modulated distinctively in SARS-CoV-2 variants. In line with the observed impaired viral kinetics of Omicron BA.1 and BA.2 variants, host immune response was induced at later stages compared to infection with other SARS-CoV-2 variants. Conclusively, infection of SARS-CoV-2 Omicron BA.1 and BA.2 variant revealed attenuated viral kinetics and deferral immune response.

### Limitations of This Study

Several Omicron subvariants with distinct properties have been described since the emergence of Omicron BA.1 in late 2021 (https://www.who.int/en/activities/tracking-SARS-CoV-2-variants. Accessed 18 September, 2022). In this study, we only analyzed BA.1 and BA.2, since other variants had not been available. Therefore, we cannot expand our findings to the currently dominating Omicron subvariants without future analyses. Additional studies will be required to transfer our findings to different primary cells and tissues, which have been shown to show differences in host cell responses (48).

## Data availability

The LC-MS/MS proteomics data have been deposited to the ProteomeXchange Consortium *via* the PRIDE partner repository with the dataset identifier PXD037265 reviewer_pxd037265@ebi.ac.uk.

## Supplemental data

This article contains supplemental data.

## Conflict of interest

The authors declare no competing interests.

## Figures and Tables

**Supplementary Figure 4:**