# Supplementary figures and images for: SARS-CoV-2 Variants Show Different Host Cell Proteome Profiles With Delayed Immune Response Activation in Omicron-Infected Cells
Source: Mol Cell Proteomics. 2023 Mar 30;22(5):100537. doi: 10.1016/j.mcpro.2023.100537 (PMC10060015; doi:10.1016/j.mcpro.2023.100537)

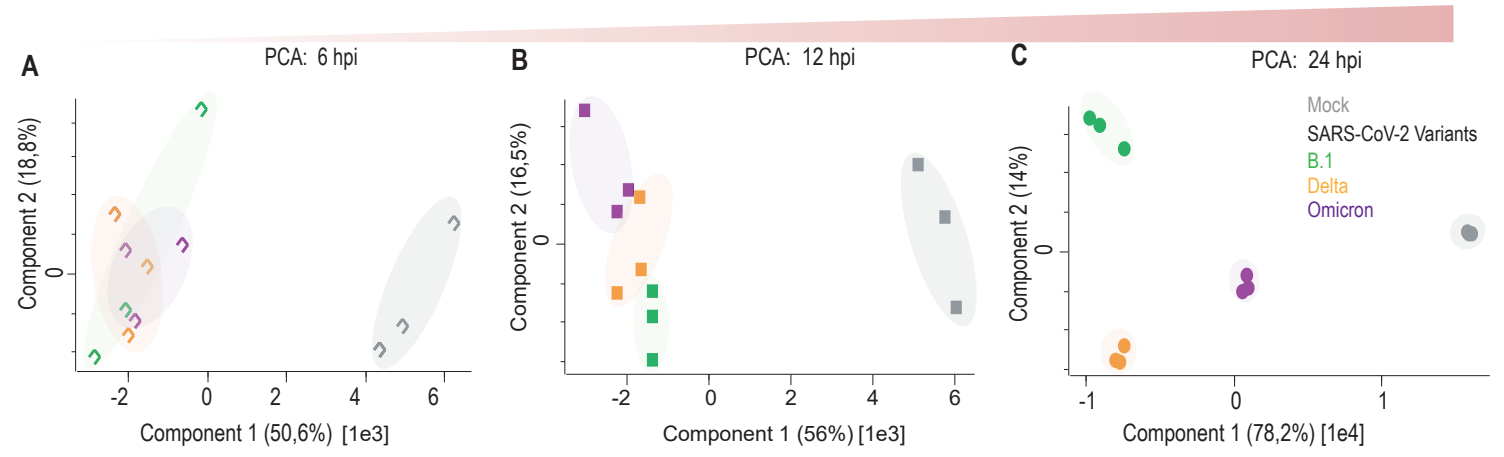

**Supp. Figure 1**

Supplement: Supplementary Figure 1 [file mmc1.pdf]

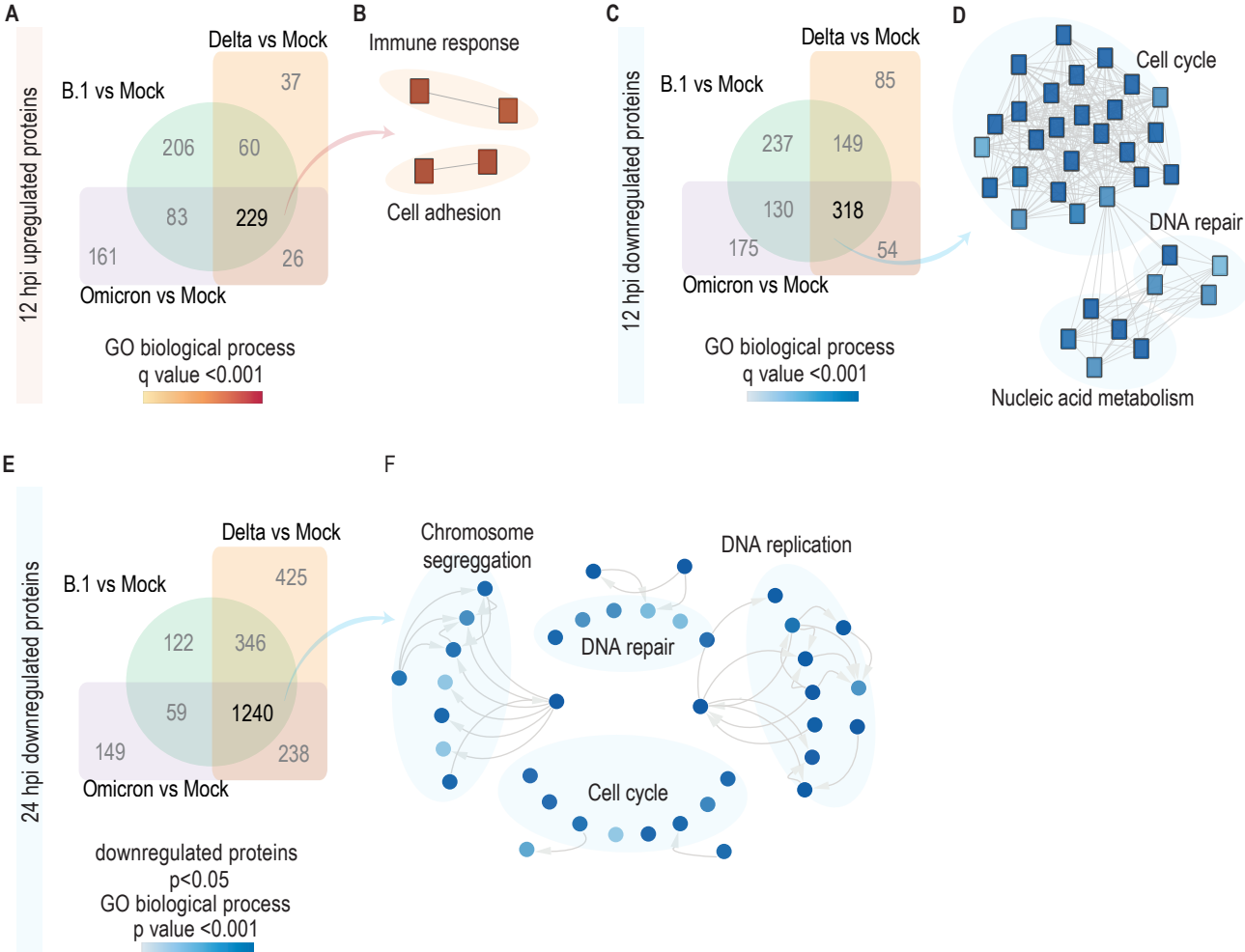

**Supp Figure 2**

Supplement: Supplementary Figure 2 [file mmc2.pdf]

**A**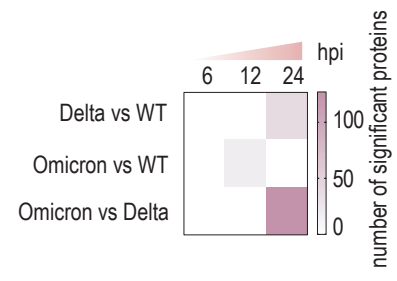**B**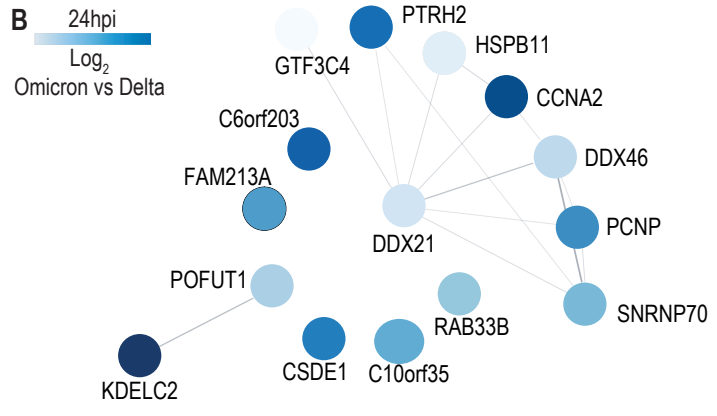**C**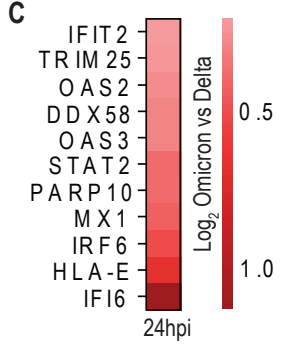**Supp Figure 3**

Supplement: Supplementary Figure 3 [file mmc3.pdf]

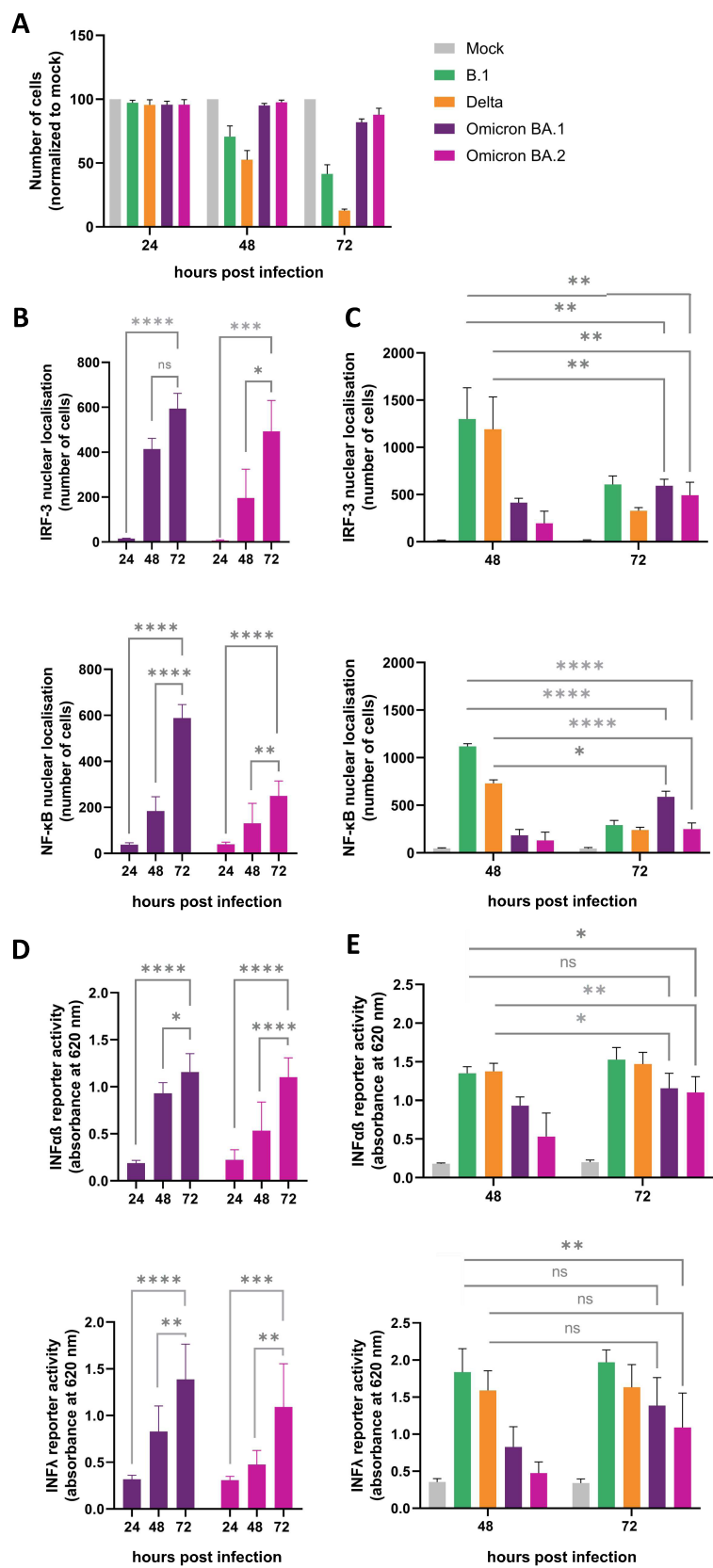

Supp Figure 5

Supplement: Supplementary Figure 5 [file mmc4.pdf]
