# Supplementary material for: SARS-CoV-2 Variants Show Different Host Cell Proteome Profiles With Delayed Immune Response Activation in Omicron-Infected Cells
Source: Mol Cell Proteomics. 2023 Mar 30;22(5):100537. doi: 10.1016/j.mcpro.2023.100537 (PMC10060015; doi:10.1016/j.mcpro.2023.100537)
Supplement: Supplementary Legends [file mmc5.docx]

**Legends**

**Supplementary Figures**

**Supplementary Figure 1: Proteomic analysis of non-infected and infected cells**

(A-C) Principal Component Analysis (PCA) of most variable differentially expressed proteins identified in total proteome analysis of mock or SARS-CoV-2 variants B.1, Delta, Omicron BA.1 infected Calu-3 cells at (A) 6, (B) 12 and (C) 24 hpi. (n=3 biologically independent replicates)

**Supplementary Figure 2: Commonly regulated pathways upon SARS-CoV-2 variant infection**

(A-D) Overlap of significantly (*q*-value ≤ 0.05) (A) increased (red) and (C) decreased (blue) proteins at 12 hpi in Calu-3 cells infected with SARS-CoV-2 variants B.1, Delta or Omicron BA.1 in comparison to mock and respective (B and D) functional gene ontology enrichment analysis (FDR *q*-value ≤ 0.001) of overlapping proteins. (E) Overlap of significantly (*q*-value ≤ 0.05) decreased (blue) proteins at 24 hpi and respective (F) GO biological process (*p*-value ≤ 0.001).

**Supplementary Figure 3: Omicron vs Delta variant proteome comparison**

(A) Significantly regulated (q-value ≤ 0.05) host cell proteins between SARS-CoV-2 variants B.1, Delta (B.1.167.2) and Omicron (BA.1) infected Calu3 cells at 6, 12 and 24 hpi. (B) STRING enrichment analysis of protein network for significantly (*q*-value ≤ 0.05) downregulated host proteins with expression pattern representing log_2_ difference at 24hpi in comparisons Omicron (BA.1) vs Delta infected cells. (C) Heatmap representing log_2_ difference of immune host proteins at 24 hpi in comparisons Omicron (BA.1) vs Delta infected cells.

**Supplementary Figure 4: Viral** **kinetics of SARS-CoV-2 variant Omicron BA.2**

(A) Mutational landscape of SARS-CoV-2 BA.2 variant within the S protein (grey boxes: existing mutations; triangles: deletions; source: https://outbreak.info/compare-lineages?pango=Omicron&pango=BA.2%2a%20%5BOmicron%20%28BA.2.X%29%5D&pango=B.1&pango=B.1.617.2&gene=S&gene=ORF1a&gene=ORF1b&gene=ORF3a&gene=ORF6&gene=ORF7a&gene=ORF7b&gene=ORF8&gene=ORF10&threshold=75&nthresh=1&sub=false&dark=false. Accessed 11 September, 2022). (B) Representative immunofluorescence images of Calu-3 cells infected with SARS-CoV-2 variant BA.2 at MOI 1 (blue: DAPI; red: S protein; scale bar represents 200 µm). Experiments were performed in parallel with other variants (Figure 1). For comparability, Omicron BA.1 values from Figure 1 were included in (C) and (D). (C) Quantification of Omicron BA.2 infected cells based on immunofluorescence images using Harmony software (PerkinElmer). (D) Omicron BA.2 RNA kinetics measured with qPCR of the RNA-dependent RNA polymerase (RdRp). Statistics of (C) and (D) were determined by two-way ANOVA (n = 3 biologically independent replicates), bars represent the mean and error bar show ± SD. Ns - not significant, ** p < 0.01.

**Supplementary Figure 5: Interferon signalling upon infection with different SARS-CoV-2 variants**.

Calu-3 cells were infected with SARS-CoV-2 variants at a MOI of 0.01 and analysed at 24 hpi, 48 hpi and 72 hpi. (A) Total number of cells determined by immunofluorescence staining. (B) Cells were fixed and nuclear translocation of IRF-3 and NF-κB was compared individually over time for BA.1 and BA.2 or (C) compared to the variants B.1 and Delta at different time points. (D) Interferon type I and III induction was measured with HEK blue reporter cells and interferon response was compared for BA.1 and BA.2 individually or (E) with the variants B.1 and Delta at different time points. Images were evaluated with the Harmony Software (PerkinElmer). Statistics determined by two-way ANOVA (n = three biological independent replicates), bars represent the mean and error bar shows ± SD. Ns - not significant, * p < 0.05, ** p < 0.01, *** p < 0.005, **** p < 0.0001.

**Supplementary Tables**

**Supplementary Table 1: Schematic representation of TMTpro 16-plex sample labelling**

TMTpro 16-plex sample labelling of cells mock infected or infected with B.1, Delta or Omicron BA.1 at 6, 12 and 24 hpi. N=3 biologically independent replicates were processed as indicated.

**Supplementary Table 2: Whole cell proteomic data of non-infected and SARS-CoV-2 variants infected cells**

All identified proteins in total proteomics of Calu-3 cells mock infected or infected with B.1, Delta or Omicron BA.1 at 6, 12 and 24 hpi. Multiple parameters from identified proteins, including protein accession number, number of unique peptides assigned to each protein, sequence % coverage etc, were reported. Quantitation data for all identified proteins represented as abundance and normalised abundance. Refer to supplementary table 1 for labelling.

**Supplementary Table 3: SARS-CoV-2 viral proteins significant between variants at 24 hpi.**

Viral proteins detected in total proteomics analysis of cells infected with B.1, Delta or Omicron BA.1 at 24 hpi. Significant values were FDR corrected and q-value < 0.05 was considered significant (+). ns: not significant. N=3 biologically independent replicates

**Supplementary Table 4: Host proteins detected in total proteomics of SARS-CoV-2 variant infected cells compared to mock control**

Host proteins detected in total proteomics analysis of cells mock infected or infected with B.1, Delta or Omicron BA.1 at 6, 12 and 24 hpi. Log_2_ transformed value of protein abundance in individual replicates. Significance determined between variant infected cells and control mock in the respective multi-plex (MP) run. Significance determined between variants as indicated. Significant values were FDR corrected and q-value < 0.05 was considered significant (+). N=3 biologically independent replicates.

**Supplementary Table 5: Comparison of host cell proteins between SARS-CoV-2 variant infected cells**

Log_2_ transformed protein abundance value of host proteins detected in total proteomics analysis of cells infected with B.1, Delta or Omicron BA.1 at 6, 12 and 24 hpi in individual replicates. Significance determined between variants as indicated. Significant values were FDR corrected and q-value < 0.05 was considered significant (+). N=3 biologically independent replicates
