# Supplementary material for: SARS-CoV-2 Variants Show Different Host Cell Proteome Profiles With Delayed Immune Response Activation in Omicron-Infected Cells
Source: Mol Cell Proteomics. 2023 Mar 30;22(5):100537. doi: 10.1016/j.mcpro.2023.100537 (PMC10060015; doi:10.1016/j.mcpro.2023.100537)
Supplement: Supplementary Table 1 [file mmc6.pdf]

Schematic representation of TMTpro 16plex sample labelling.

Multiplex	126	127 N	127 C	128 N	128 C	129 N	129 C	130 N	130 C	131 N	131 C	132 N	132 C	133 N
**MP1: 6 hpi**	BC 1	R1 B.1	R2 B.1	R3 B.1	R1 Delta	R2 Delta	R3 Delta	R1 Omicron	R2 Omicron	R3 Omicron	R1 Mock MP1	R2 Mock MP1	R3 Mock MP1	BC 2
**MP2: 12 hpi**	BC 1	R1 B.1	R2 B.1	R3 B.1	R1 Delta	R2 Delta	R3 Delta	R1 Omicron	R2 Omicron	R3 Omicron	R1 Mock MP2	R2 Mock MP2	R3 Mock MP2	BC 2
**MP3: 24 hpi**	BC 1	R1 B.1	R2 B.1	R3 B.1	R1 Delta	R2 Delta	R3 Delta	R1 Omicron	R2 Omicron	R3 Omicron	R1 Mock MP3	R2 Mock MP3	R3 Mock MP3	BC 2

BC: Bridge Channel (equal concentrations of all samples)

MP: Multiplex

hpi: hours post infection

N=3 biologically independent replicate (R)
