# Supplementary material for: SARS-CoV-2 Variants Show Different Host Cell Proteome Profiles With Delayed Immune Response Activation in Omicron-Infected Cells
Source: Mol Cell Proteomics. 2023 Mar 30;22(5):100537. doi: 10.1016/j.mcpro.2023.100537 (PMC10060015; doi:10.1016/j.mcpro.2023.100537)
Supplement: Supplementary Table 3 [file mmc8.pdf]

SARS-CoV-2 viral proteins significant between variants at 24 hpi							
		Significance			FDR q-value		
Protein id	Gene symbol	Delta vs B.1	Omicron vs B.1	Omicron vs Delta	Delta vs B.1	Omicron vs B.1	Omicron vs Delta
P0DTC7	7a	ns	ns	ns	0,43059	0,465696	0,0727006
P0DTD2	9b	+	ns	+	0	0,253294	0,0136
P0DTC2	S	ns	ns	+	0,0561951	0,146333	0,0118261
P0DTC3	3a	ns	ns	+	0,881793	0,167333	0,02125
P0DTC6	NSP6	ns	ns	+	0,747202	0,159636	0,0126512
P0DTC5	M	ns	ns	+	0,803253	0,143429	0,0437667
P0DTC9	N	ns	ns	+	0,0729362	0,112	0,0114805
P0DTC8	NSP8	+	ns	+	0,0472727	0,125778	0,0188889
P0DTD1	REP	ns	ns	+	0,440881	0,219429	0,0100741

Viral proteins detected in whole cell proteome of cells infected with B.1, Delta (B.1.167.2) or Omicron BA.1 at 24 hpi

No significance was observed at 6 and 12 hpi and signifance determined as described in Proteomics methods.

Significant values were FDR corrected at q-value < 0.05 was considered significant (+). ns: not significant.

N=3 biologically independent replicates
